# Proteases Revisited: Roles and Therapeutic Implications in Fibrosis

**DOI:** 10.1155/2017/2570154

**Published:** 2017-05-31

**Authors:** Jakub Kryczka, Joanna Boncela

**Affiliations:** Institute of Medical Biology, Polish Academy of Sciences, Lodz, Poland

## Abstract

Proteases target many substrates, triggering changes in distinct biological processes correlated with cell migration, EMT/EndMT and fibrosis. Extracellular protease activity, demonstrated by secreted and membrane-bound protease forms, leads to ECM degradation, activation of other proteases (i.e., proteolysis of nonactive zymogens), decomposition of cell-cell junctions, release of sequestered growth factors (TGF-*β* and VEGF), activation of signal proteins and receptors, degradation of inflammatory inhibitors or inflammation-related proteins, and changes in cell mechanosensing and motility. Intracellular proteases, mainly caspases and cathepsins, modulate lysosome activity and signal transduction pathways. Herein, we discuss the current knowledge on the multidimensional impact of proteases on the development of fibrosis.

## 1. Introduction

**Figure 1:**
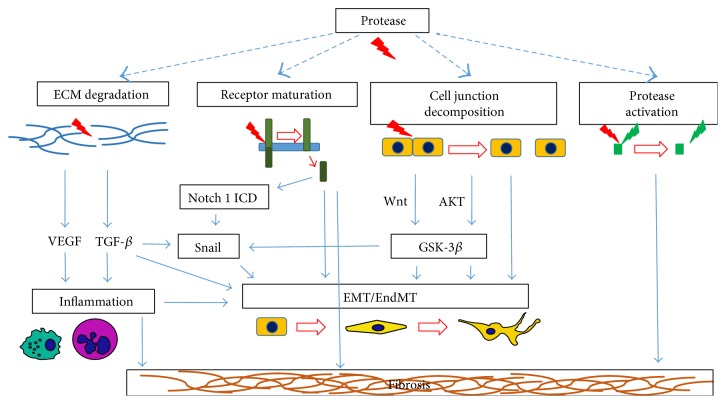
The involvement of proteases in divergent processes leading to mesenchymal transition and fibrosis. Proteolytic activity leads to cell junction decomposition and ECM degradation with liberation of sequestered growth factors such as TGF or VEGF that increase leukocytes infiltration and prolong inflammation. Furthermore, other proteases, for example, MMP from inactive zymogens and receptors from immature receptor protein are activated. Wnt and Akt signaling sustain EMT program. TGF-*β* or Notch 1-related signaling upregulates the expression of Snail transcription factor that in turn, triggers EMT program. All processes result in scar tissue accumulation and fibrosis.

Epithelial and endothelial cells establish close cell-cell contacts with a certain cell polarity, forming through desmosomes and tight and adherens junctions a solid barrier that maintains organism homeostasis. The development of fibrosis, a pathological process characterized by the increased production and deposition of extracellular matrix (ECM) components and vast accumulation of myofibroblasts, is closely related with ongoing epithelial or endothelial to mesenchymal transition (EMT or EndMT) [1]. During EMT/EndMT, cells lose their origin markers, polarity, and cell-cell connections and gain promigratory phenotypes accompanied by acquisition of mesenchymal markers [2–4]. EMT-obtained spindle-shaped mesenchymal-like cells pose high-migratory abilities. They may infiltrate into inflammatory tissue using mesenchymal or mixed mesenchymal (an amoeboid type of migration) based on protease-mediated degradation of ECM. Migration may be defined as organized cell movement in specific directions, either on top of other cells or ligands (2D) or through complex microenvironments, typically in three-dimensional (3D) fibrillar networks, triggered by certain factors [5]. 2D cell migration is characterized by a series of events that always begin with a back-to-front polarization in response to extracellular signals. The efficacy of 2D cell motility relies on highly coordinated dynamic assembly and disassembly cycles of adhesion sites from the front to the rear of the cell. The major cell surface receptors for cell adhesion to ECM structures belong to the integrin family, while the majority of proteases that are known to be involved in migration act directly or indirectly on integrin deactivation. This can occur either by direct cleavage of integrin extracellular domains or by proteolysis of ECM proteins that are integrin ligands [6–8]. However, during 3D migration, through the base of the membrane, cell layers and ECM cells form specialized structures called invadosomes that blend adhesive properties with proteolytic abilities, allowing cells to infiltrate the tissue [9–11]. Invadosomes can be divided into podosomes (short-lived, punctate, ring-shaped structures) and invadopodia (larger, longer lasting protrusions) [9, 12–18]. Independently of invadosome type, the main principle of operation remains similar. At the initial stage, a structure is formed by adhesion to ECM components via many receptors, mainly integrins, followed by clustering into phosphatidylinositol (3,4)-bisphosphate-enriched areas of the membrane. Next, phosphorylation of several proteins mediated by Src, Tks5, and Grb2 activates the Arp2/3 complex that leads to elongation and formation of columnar actin structures. Simultaneously, proteolysis of ECM components by both cell membrane-bound and cell membrane-secreted proteases begins in close vicinity of already formed adhesion hotspot. Degradation of ECM components results in decreased adhesion, forcing invadosomes' furtherer elongation of columnar structures toward increased ECM rigidity. Adhesion to its deeper layers shifts degradation of ECM components further, and by the constant and dynamic reformation of invadosomes, leading cells to cross anatomical boundaries [19, 20]. However, protease involvement in the development of EMT/EndMT and fibrosis is limited not only to ECM degradation. In general, the contribution of protease activity to fibrosis can be exerted both intracellularly and extracellularly [21]. Extracellular protease activity, demonstrated by secreted and membrane-bound protease forms, is very composed and leads to the activation of other proteases (i.e., proteolysis of nonactive zymogens), decomposition of cell-cell junctions, release of sequestered growth factors (TGF-*β* and VEGF), activation of signal proteins and receptors, degradation of inflammatory inhibitors or inflammation-related proteins, and changes in cell mechanosensing and motility. Intracellular proteases modulate lysosome activity and signal transduction pathways [21]. All in all, proteases target many substrates, thus inflicting changes in distinct biological processes correlated with cell migration, EMT/EndMT and fibrosis (Figure 1).

Even though for more than last two decades matrix metalloproteinases (MMPs) were considered to be the major targets for therapies focused on termination of cell migration (treatment of cancer and inflammation related to fibrosis or arthritis), MMP inhibitors failed to be clinically worthy, as a broad range of MMP inhibition led to severe side effects [22]. Currently, better understanding of the biological role of MMPs and their complex substrate network, consisting of not only ECM components but also cell surface receptors (i.e., integrin), cell-cell contact proteins, chemokines, and signal molecules, allowed for the assumption that other proteases may be more effective as therapeutic targets during cell migration-related disease [1, 8, 22]. Therefore, in this manuscript, we focused on proteases, other than MMPs, which are involved in the progression of EMT/EndMT and fibrosis, as they may become new markers and targets for antifibrotic therapy. We revived a group of proteases that we identified in microvascular endothelial cells (HMEC-1) treated with TGF-*β*2 Table 1. TGF-*β*2 is a potent EMT or EndMT inducer, often used as a model of naturally ongoing mesenchymal transition [2–4, 23, 24].

## 2. Cathepsins

Cathepsins (from the Greek *kathepsein*—to digest) are a family of proteolytic enzymes expressed in all organisms from plants to humans. They are active in slightly acidic environments, and all 15 members of human cathepsins can be categorized according to their localization as intracellular (lysosomal) and extracellular cathepsins and structurally by the catalytic active site residue: serine (cathepsins A and G), aspartate (cathepsins D and E), or cysteine (cathepsins B, C, F, H, K, L, O, S, V, X, and W) [25].

The activity of cathepsins is closely related with autophagy process. Autophagy is involved in control and coordination of inflammatory response and may play either profibrotic or antifibrotic role [26]. The inhibition of autophagy suppressed fibronectin accumulation and apoptosis, while the enhancement of autophagy increased TGF-*β*1-induced cell death in the mouse renal fibrosis model of unilateral ureteral obstruction (UUO) [27]. However, in the same UUO model, valproic acid- (VPA-) induced autophagy attenuated fibrotic tissue formation [28]. The antifibrotic role of autophagy was also reported during pulmonary fibrosis, since pretreatment with rapamycin (autophagy inducer) decreased the bleomycin-mediated fibrosis observed in mice [29]. In human cirrhosis liver, the increased level of cathepsin D was observed to colocalize with autophagy marker—microtubule-associated protein 1 light chain 3B (LC3B) and lysosome-associated membrane protein-1 [30]. Cirrhotic tissue present increased number of autophagosomes in comparison to normal one [30], nonetheless the number of autophagosomes was decreased in the mouse lungs after bleomycin exposure [29]. Moreover, recent findings suggest that cathepsins D, B, and L regulate the process of autophagy and lysosome-mediated protein degradation [31, 32].

To invade surrounding tissue during EMT, fibroblasts and myofibroblasts acquired from epithelial cells use the mesenchymal type of migration based on the adhesion structures such as invadosomes. Cysteine cathepsins B, X, S, L, and H, whose substrates are fibronectin, laminin, and collagen types I and IV, were reported to be involved in ECM degradation in cortacti-rich protrusion—podosomes formed in 3D Matrigel by macrophages [33] and in v-Src-transformed mouse fibroblasts [34]. Cathepsins not only degrade collagen but also, as recently suggested, take part in repression of expression of collagens III and IV by fibroblasts in a TLR2/NF-*κ*B-related manner [35]. This mechanism is believed to be responsible for prolonged wound healing and increased inflammation of damaged tissue. Moreover, cell motility was correlated with cysteine cathepsin activity. Cathepsin inhibitors CA-074, AMS-36, and LHVS significantly decrease the ability of macrophage invasion [33].

Cells during ECM decomposition can internalize its components such as collagen, through endocytosis followed by cathepsin degradation in the lysosomes, thus allowing faster cell infiltration into tissue and forcing other cells to exceed collagen production and secretion. Aberrant ECM remodeling can directly lead to fibrosis and other pathological states, such as osteoarthritis and cancer [21]. Degradation of ECM components by cathepsins does not only remove physiological barriers for migration but also releases sequestered profibrotic growth factors, such as TGF-*β*, VEGF, or PDGF [1]. These factors increase inflammation, leading in turn to fibrosis [21].

Moreover, cathepsins B, L, and S secreted from epithelial pulmonary cells are involved in the degradation of defensins (*β*-defensin family), antibacterial catalytic proteins, lactoferrins, and surfactant protein A (SP-A), decreasing antimicrobiological activity during microbiological infection, prolonging inflammation, and thus inflicting lung fibrosis [22, 36–38]. The activities of cathepsins B, L, and S are increased in bronchoalveolar lavage (BAL) fluid in patients with cystic fibrosis (CF). These cathepsins have been reported to cleave and inactivate antiprotease secretory leukoprotease inhibitor (SLIP) of neutrophil-derived elastase, shifting delicate protease/antiprotease equilibrium toward proteolytic activity and increasing neutrophil extracellular trap (NET) formation and activity [39, 40]. Proteolytic activity may also be elevated by cathepsin L that is believed to activate pro-urokinase-type plasminogen activator (pro-uPA) to its active form (uPA) [41]. Moreover, cathepsin S expression is regulated by the IRF-1 transcription factor that is suppressed by miR-31 [38]. Downregulation of miR-31 is closely related with ongoing EMT, resulting in increased cathepsin expression correlated with epithelial cells acquiring mesenchymal phenotypes [42]. Intracellularly, profibrotic protease activity is not only limited to cathepsins, and caspases represent the another important group.

## 3. Caspases

Caspases (also known as cysteine-aspartic proteases, cysteine aspartases, or cysteine-dependent aspartate-directed proteases) are the family of intracellular proteases that cleave substrates in a highly specific manner after the Asp residue in short tetrapeptide (X-X-X-Asp) motifs [43]. The effect of caspases during fibrosis is related to signal transduction pathways. The caspase cascade is an executioner of apoptosis with apoptotic signal transduction via caspase-8 (FasL-dependent pathway) or caspase-9 (FasL-independent pathway) and activation of effector caspase-3, caspase-6, and caspase-7. The importance of apoptosis in fibrosis is uncertain and may also represent an important aberration in fibrosis. Fibroblasts derived from fibrotic lung tissue (in idiopathic pulmonary fibrosis (IPF)) are more resistant to apoptosis, shifting the delicate balance toward ECM deposition. Reduced fibroblast sensitivity to apoptosis is correlated with prostaglandin (PG) E2 deficiency. Fibrotic lung fibroblasts are both resistant to apoptosis and produce less PGE2 in response to FasL than control fibroblasts. This observation indicates that the alterations in both the apoptotic and nonapoptotic functions of Fas signaling are important in the pathogenesis of IPF [44]. On the other hand, epithelial cells during fibrosis are characterized by higher apoptosis, increasing tissue damage by deposition of postfibrotic nonfunctional tissue in lung epithelial cells, alveolar macrophages, and infiltrating inflammatory cells in mouse bleomycin-induced pneumopathy model, as well as in hepatocytes during liver fibrosis [1, 45–47]. Moreover, caspases create cross talk between autophagy and apoptosis [48]. Since autophagy is consider as a strategy for cell survival via the self-degradation of proteins or organelles during nutrient deprivation, thus caspase-mediated inhibition of autophagy is often related as proapoptotic [49, 50]. Caspases cleave several human proteins from Atg family (autophagy related) [51], that is, hAtg3 is cleaved by caspase-3, caspase-6, and caspase-8 and hAtg6 (Beclin 1) by caspase-3 and caspase-6, while hAtg9, hAtg7, and hAtg4 homologues are cleaved by caspase-3 that result in autophagy inhibition and increased apoptosis [50, 52, 53]. Furthermore, caspase-1, caspase-4, and caspase-5 take part in the activation of proinflammatory necrotic cell death called pyroptosis that occurs primarily in macrophages, monocytes, and dendritic cells (DCs), as well as in various other cell types such as T cells [54, 55]. Similar to apoptosis, pyroptosis involves caspase-mediated cleavage. Upon proinflammatory signals, canonical (via caspase-1) or noncanonical (via caspase-4 and caspase-5) pathways lead to gasdermin G cleavage that promotes the formation of membrane pores [56, 57]. Proinflammatory properties of pyroptosis is related not only to the release of cytosolic content but also to the processing and release of proinflammatory and profibrotic IL-1*β* and IL-18, which have strong activity in promoting vasodilation and extravasation of immune response cells, the generation of IL-17-producing T helper cell (Th17) and the production of interferon-*γ* (IFN-*γ*) by NK (natural killer) and Th1 cells [54]. Furthermore, IL-1*β* activates Snail through NF-*κ*B, enhancing TGF-*β*2-induced EndMT in vitro in HUVEC [58] and TGF-*β*1-induced EMT in human bronchial epithelial cells and human primary mesothelial cells prolonging inflammation [59, 60].

## 4. Neutrophil Elastase

Neutrophil elastase (NE), also known as leukocyte elastase, lysosomal elastase, or medullasin, belongs to a family of serine proteases. It consists of 218 amino acid residues and, its molecular weight is 29–33 kDa. NE shares approximately 55% sequence similarity to human proteinase 3 and approximately 35% to cathepsin G [61, 62]. Neutrophil elastase is stored in primary azurophilic granules, and during ongoing inflammatory processes, it is released by neutrophils to the extracellular milieu [40, 62]. As neutrophils are the first leukocytes to appear at the site of wounded or infected tissue, the primary function of NE is to provide protection against microbiological infection [1, 63]. Neutrophil extracellular traps (NET) composed of extruded DNA in the form of decondensed chromatin coated with antimicrobial proteins, such as defensins and neutrophil elastase, entrap and utilize microbiological threads [64, 65]. In the early phase (15 min), NET are induced via autophagy or via both autophagy and reactive oxygen species (ROS) [66]. Thus, the activity and level of NEs are closely related to ongoing inflammation, and according to many reports, NE levels have been highly elevated in BAL fluid of CF patients [40]. Furthermore, this elastase induces autophagy through the upregulation of placental growth factor (PGF) which in turn promotes lung epithelial cell apoptosis and pulmonary emphysema. PGF and its downstream MAPK8 and MAPK14 signaling pathways are potential therapeutic targets for the treatment of emphysema and chronic obstructive pulmonary disease (COPD) [67]. The degradation of SLIP (i.e., by cathepsins) enhances NE proteolytic abilities resulting in increased fibrosis, by the destruction of epithelial and endothelial cell matrixes and prolonged inflammation. Active NE cleaves interstitial collagen type III (*α*1 chain Ala-Gly-Ile^779^/^∗^/Thr^780^-Gly-Arg); however, it shows no collagenolytic abilities toward fibrillar collagen type III [68]. Moreover, other ECM components, such as heparan sulfate proteoglycan, were reported to be substrates for NE [63, 69]. For many years, neutrophil elastase has been known to prolong inflammation by the degradation of complements and the release of the strong neutrophil chemoattractant, component C5a [70], as well as by the upregulation of leukocyte-recruiting interleukin IL-8 expression in surrounding cells [71]. The elevated level of IL-8 is not only responsible for leukocyte recruitment but might also trigger EMT, leading directly to fibrosis. Furthermore, IL-8 increases proliferation and survival of fibrosis-related EMT-derived fibroblasts and myofibroblasts by raising the levels of Bcl-xL:Bcl-xS and Bcl-2:Bax ratios [1, 71–73].

## 5. Neprilysin

Neutral endopeptidase, also known as neprilysin (NEP), CD10, membrane metalloendopeptidase (MME), enkephalinase, or common acute lymphoblastic leukemia antigen (CALLA), is a zinc-dependent type II integral membrane peptidase EC 3.4.24.11. NEP intra- and extracellularly degrades a variety of proteins, including bradykinin, adrenomedullin, endothelin-1, enkephalins, angiotensin II, substance P (SP), or neurotensin [74, 75] and is expressed in many different organs, including the lung, kidney, prostate, intestine, and brain. Its molecular mass differs between 90 and 110 kDa and is based on tissue-specific glycosylation [74, 76]. The upregulation of NEP correlates with ongoing EMT as tumor cells with significant expression of EMT markers, such as vimentin and *α*-smooth muscle actin (*α*SMA), and S100 proteins show increased levels of CD10 on the cell surface (94% atypical fibroxanthoma, 50% of squamous cell carcinoma, and 33% of spindle cell/desmoplastic melanomas) [77, 78]. Some recent data suggest ADAM-17-dependent exosome-based release of soluble, circulating NEP [79]. Furthermore, soluble NEP is believed to be a poor therapeutic prognostic for patients with fibrosis-related heart failure (HF), as was observed in a group of patients with HF who were followed through more than 4 years of treatment [80]. Moreover, due to the degradation of vasodilatory peptides (i.e., bradykinin, adrenomedullin, endothelin-1, and angiotensin II), which leads to the dysregulation of natriuresis vasodilatation, high expression of CD10 is correlated with the loss of heart and kidney function, caused by ongoing fibrosis. Therefore, the combined inhibition of NEP and angiotensin receptors by LCZ696 (consisting of NEP inhibitor prodrug AHU337 that is cleaved into the active form LBQ657 and valsartan, an angiotensin II receptor antagonist) has been implemented as potentially antifibrotic therapy during heart and kidney fibrosis [80–83]. Furthermore, LCZ696 attenuates angiotensin-II-mediated renal cellular collagen synthesis and fibrosis development [82]. On the other hand, CD10-mediated SP degradation is believed to be involved in inhibition of skin inflammation, also demonstrating certain antifibrotic properties [84].

## 6. Presenilin-1

Presenilins are transmembrane proteases that in humans are represented by two homologs, presenilin-1 (PS-1) and presenilin-2 (PS-2), encoded by two gens, *PSEN1* and *PSEN2,* respectively [85]. The sequence of PS-1 consists of nine transmembrane helices (transmembrane domains—TMs), connected either by short or long loops, located on both sides of the cell membrane [86, 87]. Presenilin-1 is one of 4 core components of the *γ*-secretase protein complex along with nicastrin (NCT), presenilin enhancer 2 (PEN2), and anterior pharynx-defective 1 (APH1). PS-1 forms its catalytic (proteolytic) subunit, providing the degradation of many cell membrane-associated proteins, mainly adhesion or junction proteins, including CD44, N-cadherin, E-cadherin, and nectin-1 [86]. Furthermore, overexpression and activity of PS-1 associated with *γ*-secretase are critically involved in acquiring and the maintenance of mesenchymal phenotypes achieved by EMT. The inhibition of its activity by DAPT, a *γ*-secretase inhibitor, results in downregulation of EMT-related proteins, such as Snail, *α*SMA, Notch 1, COX2, and cyclin D1, and inhibition of cell mesenchymal transition and motility [88]. PS-1 cleaves membrane-bound E-cadherin, disassembles the adherent junctions, and releases pro-oncogenic and profibrotic soluble, N-terminal 80 kDa fragment known as sE-CAD that in turn, through the sustained activation of the AKT pathway, triggers EMT [89, 90]. Furthermore, the cleaved C-terminal 33 kDa intracellular fragment disassociates and releases *β*-catenin that was sequestered in the E-cadherin/*β*-catenin complex. Released into the cytosol, free *β*-catenin translocates to the nucleus and activates Wnt signaling pathways, upregulates Snail expression, and triggers EMT [88, 91, 92]. However, the results from the study related to Alzheimer's disease progression indicate that PS-1 serves additional *γ*-secretase-independent roles in Wnt signaling, as well as in lysosomal function and autophagy [93–96]. PS-1 acts as a pro-EMT, also by the maturation and activation of the transcription regulator Notch. Its activation appears to be by proteolysis in the S3 cleavage site of the Notch 1 membrane receptor and the release of the Notch 1 intracellular domain (Notch 1/ICD or NICD) [97, 98]. Next, Notch 1/ICD directly upregulates Snail (Snail-1) and Slug (Snail-2) expression, respectively, by interaction with its promoter. Moreover, NICD mediates the induction of the HIF-1*α* factor that might upregulate the expression of lysyl oxidase which stabilizes Snail-1 protein [99–102].

## 7. Urokinase

The urokinase plasminogen activator (uPA) is a serine protease that binds to its cell surface receptor (urokinase plasminogen activator receptor (uPAR)) and, after activation, is mediated by its proteolytic abilities and many biological activities (plasminogen activation, ECM remodeling, growth factors activation, and intracellular signaling initiation) [103, 104]. Urokinase structure consists of three conserved domains: (1) a growth factor-like domain (GFD, residues 1–49), (2) a kringle domain (residues 50–131), forming modular amino-terminal fragments (ATF by which uPA binds to uPAR) linked by the “connecting peptide” (CP, residues 132–158), and (3) a serine protease domain (residues 159–411) [103, 104]. The upregulation of uPA and its activity occurs during EMT and EMT-related fibrosis; however, its role is yet not clearly understood [105, 106]. First of all, extracellularly bounded to its receptor, uPA cleaves plasminogen and releases active, multipotent serine protease plasmin that in turn, mediates ECM degradation and by proteolytic cleavage of latency-associated peptide activation of TGF-*β* as well as MMPs from inactive zymogens [107–110]. Simultaneous silencing of uPA and MMP9 resulted in decreased ECM degradation and cell migration increasing adhesive capacity of the MDA-MB-231 breast cancer cells. Furthermore, the EMT-obtained mesenchymal phenotype was reversed, presenting significant downregulation of EMT-related proteins Snail and vimentin, with a simultaneous increase of epithelial phenotype marker E-cadherin protein [111]. Binding of uPA to uPAR in some certain cell types, by its intrinsic chemotactic activity, triggers signal cascades leading to increase cell motility [108].

The activities of uPA/plasmin and plasmin-dependent MMPs rely mostly on the activity of a potent inhibitor of uPA, plasminogen activator inhibitor-1 (PAI-1). Thus, by impairing the plasminogen activating systems, PAI-1 is involved in cellular proteolytic degradation of ECM proteins and the maintenance of tissue homeostasis. Whether PAI-1 is a mediator or inhibitor of fibrosis is still controversial [107]. Multiple studies using models of liver, lung, and kidney fibrosis suggest that PAI-1 deficiency or the inhibition of PAI-1 activity attenuates fibrosis. Nevertheless, homozygous deficiency of PAI-1 promotes age-dependent spontaneous cardiac fibrosis in mice, suggesting a protective role for PAI-1 in the heart [112].

## 8. Dipeptidyl Peptidase-4

Dipeptidyl peptidase-4 (DPPIV, adenosine deaminase complexing protein 2 (ADCP2), CD26) is a multifunctional serine peptidase that selectively removes the N-terminal dipeptide from peptides with proline or alanine in the second position [113]. DPPIV belongs to subfamily 9b that has a unique catalytic triad in the order of Ser, Asp, and His located in an *α*/*β*-hydrolase fold compared to the chymotrypsin catalytic triad of His, Asp, and Ser [114]. The main noncatalytic physiological function of CD26 is T cell activation by interaction with adenosine deaminase (ADA), caveolin-1, CARMA-1, CD45, mannose-6-phosphate/insulin growth factor-II receptor (M6P/IGFII-R), and C-X-C motif receptor 4 (CXC-R4). Furthermore, DPPIV also modulates the bioactivity of several chemokines [114], regulates plasma levels of the insulinotropic, glucagon-like peptide-1 hormone [115], and interacts with ECM proteins fibronectin and collagen [113]. CD26 is anchored on T lymphocytes and many endothelial and epithelial cells; however, active soluble form sCD26 (or sDPPIV) has also been reported to be present in several biological fluids (such as serum, plasma, semen, urine, synovial, and cerebrospinal fluids) [116–118]. Furthermore, in vitro studies showed sCD26 in cell medium obtained from cervical cancer cells and keratinocytes [116, 117].

Profibrotic abilities of CD26 are mainly correlated with prolonged inflammation and leukocyte maturation, as well as with increased cell migration. DPPIV is responsible for gelatin binding and ECM degradation in adhesion hotspots. DPPIV was found to colocalize with fibroblast activation protein *α* (FAP), matrix metalloproteinase (MMP2 and MMP9), urokinase plasminogen activator, and type II transmembrane serine protease, forming gelatinolytic machinery in invadopodia-like protrusions [119]. Monoclonal antibodies against the gelatin-binding domain of DPP4 blocked its ECM degradation abilities, leading to decreased migration and invasion of HUVECs [119]. However, the inhibition of CD26 activity by sitagliptin resulted in increased migration of SiHa cells [116], suggesting that its proteolytic abilities against ECM may be insufficient [116]. Moreover, the inhibition of CD26 activity by a variety of inhibitors decreases fibrosis and fibrosis-related syndromes (i.e., DA-1229) and exerted its renoprotective effect by decreasing macrophage infiltration into the kidney, thus preventing the ongoing inflammation and renal fibrosis, in mouse models. However, this mechanism remains elusive and is not well understood [120].

## 9. Therapeutic Implications

Fibrotic process results in extensive alteration of the structure and function of the certain organs; thus, clinical trials of fibrosis treatment are being conducted in many leading research facilities [121]. Antifibrotic therapies can be divided into nonpharmacological and pharmacological [121]. Because fibrosis is irreversible, nonpharmacological fibrosis therapies are based mainly on surgical restoration of organ function, either by removal of nonfunctional fibrotic scar tissue or by transplantation of whole organs, for example, lung transplantation in progressive pulmonary disease [121] or cystic fibrosis [122]. On the other hand, pharmacological therapies targeting many distinct molecular mechanisms involved in fibrosis development and progression comprise the suppression of inflammatory response by corticosteroids [123, 124], inhibition of TGF-*β*, TNF, PDGF, VEGFR, and FGFR signaling pathway, and collagen fibrils formation by tyrosine kinase inhibitor—nintedanib as well as by pyridone derivative—pirfenidone [125–127] in idiopathic pulmonary fibrosis. Furthermore, pirfenidone is being tested in other fibrosis-related disease like nonalcoholic steatohepatitis leading to cirrhosis [128], skin fibrosis (model of sclerodermatous chronic graft-versus-host disease) [129], and postsurgical excessive scarring [130]. Over the last few years, antiproteolytic approach in antifibrotic therapies was restricted mainly to matrix metalloproteinases activity; however, broad range MMP inhibitors like marimastat and prinomastat failed clinical tests, as they led to severe side effects [21, 22]. Furthermore, the role of MMPs is very complicated, as during early stages of fibrosis activity of MMP-2 and MMP-9 play profibrotic function (allowing fast accumulation of fibroblasts and leukocytes) [1] that shifts, in late stages of fibrosis, toward antifibrotic, during the degradation of massive amounts of deposited ECM as shown in skin fibrosis and keloids treatment [1, 131, 132]. It emerged over the last decade that proteases involved in blood coagulation are potential targets for therapeutic interventions in treating several fibrotic disorders. As coagulation factors seem to exert their profibrotic properties through the activation of protease-activated receptors (PARs), targeting PARs may be a more efficient (and safe) approach for limiting fibrosis. Recently, it was shown that the inhibition of PAR-2 may offer promise for potential therapy in idiopathic pulmonary fibrosis [133]. The airways of cystic fibrosis (CF) patients are characterized by neutrophils that release high amounts of elastase overwhelming the local antiprotease protection. Based on this protease/antiprotease imbalance concept, the therapeutic approaches have been developed to inhibit the elastolytic activity, including small synthetic chemical inhibitors and natural inhibitors of free elastase. The inhalation of alpha1-proteinase inhibitor (alpha1-PI), which inhibits NE activity, has been proposed as a therapeutic strategy in CF [134, 135]. The inhaled alpha-1 hydrophobic chromatography process (HC), an aerosolized alpha_1_-PI formulation, was tested. A 3-week phase 2a study confirmed that alpha-1 HC inhalation was safe and well tolerated in patient with CF [136]. Likewise, a small molecule NE inhibitor, KRP-109, inhibited mucin degradation in CF patients decreasing profibrotic protease/antiprotease imbalance. [137]. Early stages of antiapoptotic approach to antifibrotic therapy showed that inhibitor of caspases, emricasan (IDN-6556), by its caspase-3 and caspase-8 activity inhibition, decreased liver fibrosis in a murine model of nonalcoholic steatohepatitis [138]. However, further data needs to be obtained, as FK506, a generally applied immunosuppressant in organ transplantation and promotor of nerve regeneration, reduced scar formation after sciatic nerve injury in rats by inducing fibroblast apoptosis [139]. Although, several cathepsin inhibitors (e.g., CA-074Me and pepstatin A) proved antifibrotic properties in models of rat and murine kidney fibrosis [140, 141], and nothing is currently known about the clinical disposition of any of the cathepsin inhibitors discovered so far. This point suggests that there is still a lot of work to do in the design of stable, pharmacologically active compounds to be able to specifically regulate the in vivo activity of cathepsins [142]. Nevertheless, new clinical targets may emerge from the discovery of many, so far unknown, profibrotic functions of proteases.

## 10. Conclusion

**Figure 2:**
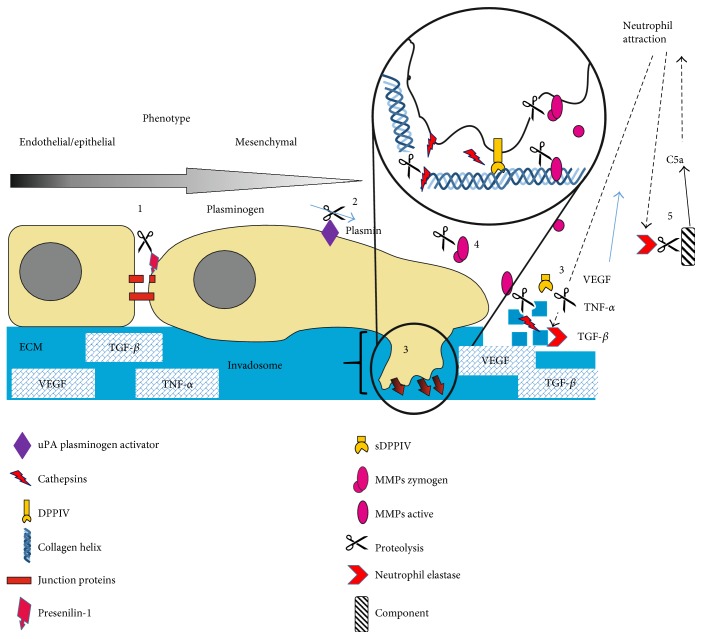
Extracellular protease activity during mesenchymal transition and fibrosis, as a potential therapeutic target. (1) Cell-cell junction decomposition. (2) uPA cleaves plasminogen and unlocks active, multipotent serine protease plasmin. (3) ECM degradation via invadosome and by secreted proteases. Release of sequestered growth factors. (4) MMP's activation via proteolysis of inactive zymogens. (5) Release of the strong neutrophil chemoattractant—complement component C5a.

**Figure 3:**
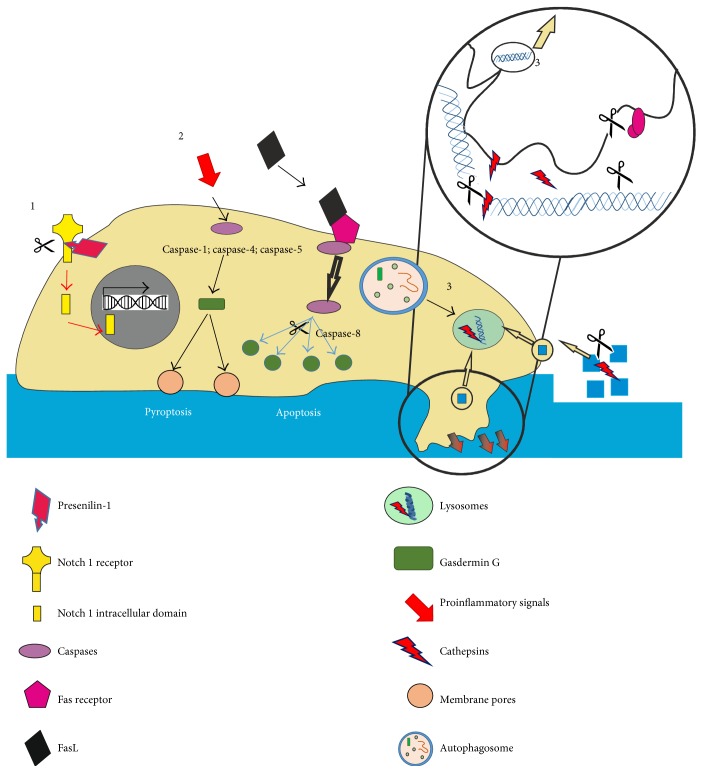
Intracellular protease activity during mesenchymal transition and fibrosis. (1) Snail1 upregulation via Notch 1 intracellular domain signaling pathway triggered by presenilin-1. (2) Caspase-mediated cell death via pyroptosis (necrosis) or apoptosis. (3) Cathepsin-mediated degradation of ECM components in lysosymes.

Fibrosis is a very complex pathological organism response, causing massive and uncontrolled deposition of scar tissue in the affected organs such as the heart, liver, kidney, lungs, or skin. Furthermore, fibrotic tissue does not show properties of the tissue it originates from, leading to systematic loss of organ function and death [1]. Fibrosis might be triggered by a variety of different factors from physical, chemical, or mechanical irritation through disrupted, uncontrolled tissue regeneration and prolonged inflammation due to autoimmune or cancer-associated signaling. Although triggering mechanisms may differ, ongoing fibrosis appears to develop according to similar mechanisms, resembling molecular mechanisms of wound healing and is sometimes referred to as unhealed wounds [143–145]. During the first crucial steps, cells migrate toward fibrotic-unhealed wounds through solid barriers composed of ECM and epithelial or/and endothelial cells. This process is strictly correlated with the degradation and processing of ECM and adherent junction proteins. Even though three classes of matrix-degrading enzymes could be responsible for invadosome-correlated motility (MMPs, cathepsin cysteine proteases, and serine proteases), zinc-dependent matrix metalloproteinases (MMP2, MMP9, and MMP14) were mainly considered as the key proteases involved in this process [10, 146]. However, recent data and the failure of inhibition of MMPs as therapeutic approaches suggest that many other proteases are involved in invadosome activity (either by ECM degradation, MMP activation or ECM adhesion, and complex stabilization) or may partially take over the role of MMPs. Unfortunately, a biology of invadosome formation, as well as activation and protease involvement, is yet not fully understood, leaving more questions than answers [146]. Furthermore, increased migration of cells ongoing EMT/EndMT, correlated with invadosome activity, is not the only critical role of proteases in fibrosis development. During the processing of ECM, many sequestered cytokines and growth factors, such as TGF-*β* or TNF-*α*, are released, leading to Snail upregulation and EMT [24, 91, 147]. Moreover, interleukins and growth factors, involved in leukocyte recruitment and activation, lead to prolonged inflammation that expose affected tissue to profibrotic factors, cell damage and ECM secretion, and effectively increase the chances of massive scar tissue deposition [1, 63, 148, 149]. Extracellular and intracellular profibrotic activities of reviewed proteases are summarized in Figures 2 and 3.

The upregulation of non-MMP proteases is curtailed during fibrosis development, suggesting their potential role as markers and therapeutic targets; however, they need to be further investigated.

## Figures and Tables

**Table 1:** The increase in protease level in microvascular endothelial cells (HMEC-1) during EndMT.

Protease name	Cell lysates	Cell medium
Presenilin	xxxx	----
Neprilysin (CD10)	xxx	----
Cathepsin C	xxx	x
Cathepsin S	xxx	x
Cathepsin V	xx	----
Cathepsin X/Z/P	xx	----
uPA	xx	x
DPPIV (CD26)	----	xx

Proteases are the main enzymes implicated in ECM organization and remodeling. To examine the role of the proteases, other than MMPs, during EndMT in microvascular endothelial cells, we compered the protease protein profile in HMEC-1 versus HMEC-1 treated with TGF-*β* for 24 h using the proteome profiler antibody array (R&D System, ARY025). In our experiments, we analysed both cell lysates and cell medium to establish protease expression and secretion level, respectively. x indicates upregulation intensity. “----” indicates no changes in intensity between HMEC-1 and HMEC-1 treated with TGF-*β*.
